# Potato Starch-Based Film Incorporated with Tea Polyphenols and Its Application in Fruit Packaging

**DOI:** 10.3390/polym15030588

**Published:** 2023-01-24

**Authors:** Nan Chen, Hao-Xiang Gao, Qiang He, Wei-Cai Zeng

**Affiliations:** 1 Antioxidant Polyphenols Team, Department of Food Engineering, Sichuan University, Chengdu 610065, China; 2 The Key Laboratory of Food Science and Technology of Sichuan Province of Education, Sichuan University, Chengdu 610065, China

**Keywords:** tea polyphenols, potato starch, composite film, antioxidant activity, fruit packaging

## Abstract

Effects of tea polyphenols (TP) on the physical properties, barrier properties and functionality of potato starch-based film were determined, while the interaction mechanism between TP and starch in film and the application of this film in fruit packaging were further evaluated. TP exhibited different effects on the physical properties of potato starch-based film, including thickness (0.083 to 0.087 mm), moisture content (9.27% to 9.68%), color (Δ*E* value: 5.41 to 10.55), light transmittance (51% to 62%), tensile properties and thermal properties, and improved its barrier properties, including water vapor permeability (9.68 to 11.84 × 10**^−^**^11^ g m**^−^**^1^ s**^−^**^1^ Pa**^−^**^1^),oxygen permeability (1.25 to 2.78 × 10**^−^**^16^ g m**^−^**^1^ s^−1^ Pa**^−^**^1^) and antioxidant activity. According to the determination of wide-angle X-ray diffraction, Fourier transform infrared and scanning electron microscope, TP could interact with starch chains via hydrogen bonds to form non-crystal complexes, thus affecting the cross-linking among starch chains and further changing the microstructure of film. Furthermore, film incorporated with TP could improve the storage quality (including weight and texture) of blueberries, and inhibit the enzymatic browning of fresh-cut bananas during storage. All present results suggested that tea polyphenols had potential to enhance the properties and function of potato starch-based film, and the film exhibited the application prospect in fruit packaging and preservation.

## 1. Introduction

With the increase of environmental consciousness and consumption levels, more and more researches are focused on the renewable and biodegradable packaging substances to replace the petrochemical-based packaging film and non-biodegradable materials [1,2]. Among these packaging materials, polysaccharides (such as starch, cellulose, chitosan and so on) are found abundantly in nature and have strong film-forming capacities [3,4]. Besides, starch exhibits great potential to prepare edible and biodegradable films for prolonging the shelf life of food (especially for fruit, vegetables and meat), due to its convenient sources, low price, good biocompatibility, innocuity and no pollution to environment [5,6]. However, some disadvantages of starchy film, such as high aging temperature, inadequate functionality, weak physical and barrier properties, restricts its widespread usage as a thermoplastic polymer film [7].

Polyphenols are ubiquitously found in cereal grains, fruits and vegetables, which are natural and renewable resources [8]. Many studies have shown that polyphenols exhibit multiple biological functions, such as antioxidant, anti-bacterial, anti-viral, etc. [8,9]. Besides, it has been reported that some polyphenols can modify the physicochemical properties (such as rheology, crystallinity and gel structure) and induce the gelatinization of starch [9,10]. Meanwhile, incorporating polyphenols into edible film is an alternative approach to fortify the effects of film for food packaging. Therefore, polyphenols are proper to incorporate with starch for improving the quality of starch-based film, so as to develop the bioactive and intelligent packaging.

Tea polyphenols (TP), a general name of phenolic compounds in tea, is the main functional component of tea. In fact, TP is the mixture of compounds with multiple hydroxyl groups in tea, and its main constituents are catechins, including epicatechin (EC), epigallocatechin (EGC), epicatechin gallate (ECG) and epigallocatechin gallate (EGCG) [11]. It has been reported that TP exhibits a variety of beneficial effects for human health, such as antioxidant, antiradiation, antimicrobial, antitumor and antithrombotic activities [12]. In light of the biological functions of TP, TP may be incorporated into starch-based film to improve its properties and functions for food packaging, especially for the fruits vulnerable to oxidative damage.

The aim of this study was to evaluate the effect of TP on the physical properties (including thickness, moisture content, color, light transmittance, tensile properties and thermal properties), barrier properties (water vapor permeability and oxygen permeability) and functionality (antioxidant activity) of potato starch-based film. Meanwhile, the interaction mechanism between TP and starch were explored by using multispectral methods. Furthermore, blueberries and fresh cut bananas were adopted to evaluate the application of starch-TP composite film in fruit packaging.

## 2. Materials and Methods

### 2.1. Materials and Reagents

Potato starch (containing 19.8% of amylose and 80.2% of amylopectin), sodium carboxymethylcellulose (CMC, purity ≥ 98%), konjac glucomannan (KGM, purity ≥ 99%) and tea polyphenols (TP, purity ≥ 98%) were purchased from Aladdin (Shanghai, China). Blueberries and bananas were purchased form a supermarket in Chengdu. All other reagents used were of analytical grade, and the water was purified by a UPR-II-10T pure water instrument (ULUPURE, Chengdu, China).

### 2.2. Preparation of Potato Starch-Based Film Incorporated with Tea Polyphenols

Briefly, potato starch (4 g), glycerol (1.2 g), CMC (0.2 g), KGM (0.1 g) and calcium chloride (0.05 g) were mixed with distilled water (100 mL). After that, tea polyphenols (TP) were added in the suspension to reach the final concentration of 2.5%, 5.0% and 7.5% (*w*/*w*, based on the weight of potato starch in the suspension), respectively. Subsequently, the suspension was heated with constant stirring (300 r/min) in a boiling water bath for 30 min; then, every 10 mL of the starch film-forming solution was poured into the polytetrafluoroethylene round petri dish (70 mm in diameter). Finally, all dishes were dried at 45 °C for 36 h, and then balanced at 25 °C under a relative humidity of 55% for 48 h before further tests [13].

### 2.3. Determination of the Physical Properties (Thickness, Moisture Content, Color, Light Transmittance, Tensile Properties and Thermal Properties) of Potato Starch-Based Film Incorporated with TP

According to the previous study [3], the thickness was determined with a ACE101-25 digital micrometer (Dongguan quick measuring instrument Co., Ltd., Dongguan, China). The moisture content was measured by the direct drying method. The color was measured with a colorimeter (Konica Minolta, Chroma Meter, CR400, Tokyo, Japan) and the film without TP was set as control. The Δ*E* value was calculated as: Δ*E* = [(L* **−** *L*_0_)^2^ + (a* **−** *a*_0_)^2^ + (b* **−** *b*_0_)^2^]^1/2^, where L*, a* and b* are the color parameters of film incorporated with TP and *L*_0_, *a*_0_ and *b*_0_ are the color parameters of control.

Light transmittance of film was measured according to the previous study with an ultraviolet-visible spectrophotometer (UV-1800PC, MAPADA, Shanghai, China). Briefly, the film was cut into a rectangular strip (40 mm × 10 mm); then, the absorbance spectrum of film was measured in the range of 400 to 800 nm [14].

Tensile properties of film, including the tensile strength (TS) and elongation at break (EB), were determined with a TA-XT express texture analyzer (TA-XT2, Stable Micro Systems Ltd., Haslemere, UK) with an A/SPR probe. Prior to the test, the film was cut into a rectangular strip (50 mm × 10 mm) and fixed between two tensile grips. The initial grip separation was set at 40 mm and the stretching speed was 3 mm/s [3].

Thermal properties of film was analyzed using a NETZSCH-STA449C TG/DSC simultaneous thermal analyzer (NETZSCH Ltd., Selb, Germany). Briefly, 5 mg of the lyophilized film was pulverized and put into an aluminum crucible. Thereafter, the aluminum crucible with sample was incubated at 30 °C for 1 h, and then heated from 30 to 500 °C at a rate of 10 °C/min with a nitrogen flow rate of 20 mL/min. Thermogravimetric (TG) curve was recorded, and the first derivative of TG curve was defined as a DTG curve [15]. 

### 2.4. Determination of the Barrier Properties (Water Vapor Permeability and Oxygen Permeability) of Potato Starch-Based Film Incorporated with TP

Water vapor permeability (WVP) of the film was measured according to the previous study. Briefly, anhydrous calcium chloride (10 g) was put into a glass jar to achieve 0% relative humidity. After covering with film sample, the glass jar was moved into a desiccator containing the saturated sodium chloride solution (25 °C, 75% of relative humidity). Then, the weight of glass jar was recorded at 0, 2, 4, 8, 12, 24, 36, 60 h, respectively. WVP value of the film was calculated as: WVP (g·m^−1^·s^−1^·Pa^−1^) = (*m* × *n*)/(*a* × *t* × Δ*p*), where *m* is the change in jar weight (g), *n* is the thickness of film (m), *a* is the opening area of the jar mouth (m^2^), *t* is the placement time (s) and Δ*p* is the partial pressure difference existed between the two sides of film sample (Pa) [16].

Oxygen permeability (OP) of the film was evaluated by the oxidative dehydrogenation degree of ascorbic acid, which was determined according to 2,6-dichloroindophenol titration method [17]. Prior to the test, 1 mL of ascorbic acid solution (0.5 mg/mL) and 10 mL of metaphosphoric acid solution (20 mg/mL) were added into a glass jar. Then, the glass jar mouth was covered with film sample and moved into a box with 70% oxygen concentration atmosphere for 24 h. After that, the solution in glass jar was titrated with 2,6-dichloroindophenol titration solution to just pink, according to the detailed method in previous study. OP value of the film was calculated as: OP (g·m^−1^·s^−1^·Pa^−1^) = (*m* × *n*)/(*a* × *t* × Δ*p*), where *m* is the content of oxidized ascorbic acid in jar (g), *n* is the thickness of film (m), *a* is the opening area of the jar mouth (m^2^), *t* is the placement time (s), and Δ*p* is the partial pressure difference between the two sides of film sample (Pa) [16].

### 2.5. Determination of the Antioxidant Activity of Potato Starch-Based Film Incorporated with TP

Briefly, film was cut into pieces, and 0.1 g of pieces was immersed in distilled water (20 mL) and then incubated at 25 °C with constant stirring for 24 h. Thereafter, the supernatants were collected and used for ABTS (2, 2’-azinobis-3-ethylbenzthiazoline-6-sulphonateand), DPPH (1,1-diphenyl-2-picrylhydrazyl) free radical scavenging assays and FRAP (ferric reducing antioxidant power) assay, respectively. All the detailed methods were described in our previous study [18].

### 2.6. Determination of Wide-Angle X-ray Diffraction (WAXD) Pattern of Potato Starch-Based Film Incorporated with TP

The WAXD pattern of film was determined by using a wide-angle X-ray diffractometer (D8 Advance, Bruker, Ltd., Berlin, German) operating at 40 kV and 40 mA with CuKα radiation (λ = 0.154 nm). The diffraction angle was from 5° (2θ) to 40° (2θ), the scanning speed was 4°/min and the step size was 0.02° [19].

### 2.7. Fourier Transform Infrared (FTIR) Spectrum Analysis of Potato Starch-Based Film Incorporated with TP

The film was analyzed by a FTIR spectrometer (Perkin Elmer, CA, USA). Prior to the test, 1 mg of lyophilized film was mixed with 100 mg of dry potassium bromide, and then ground and squashed. After that, the resulting powder was moved into a compression mold and pressed under vacuum into tablets. In detail, the scan range was 4000**–**400 cm**^−^**^1^, scan number was 32 and resolution was 2 cm**^−^**^1^.

### 2.8. Scanning Electron Microscope (SEM) Observation of Potato Starch-Based Film Incorporated with TP

The surface microstructure of film was observed with a scanning electron microscope (SEM, SU8010, Hitachi, Ltd., Tokyo, Japan) at an accelerating voltage of 3 kV within the magnification of 500 and 1000 times. Prior to the test, film was cut into a rectangular strip (1 cm × 1 cm) and then the surface of the testing sample was exposed to gold sputtering.

### 2.9. Fruit Packaging with Potato Starch-Based Film Incorporated with TP

#### 2.9.1. Packaging of Blueberries and Fresh-Cut Bananas

Blueberries and bananas of the same maturity level and size with no mechanical damage were selected for the test. (1) Blueberries were placed in a glass jar, then the glass jar mouth was covered with film and stored at 25 °C under a relative humidity of 55% for 1, 4 and 7 d before further tests. (2) Whole banana was manually cut in five pieces. Cutting boards, utensils and containers were sanitized to minimize contamination of microorganisms. After cutting, the banana pieces were quickly placed into sterile glass dishes; then, each dish mouth was covered with film, and all dishes were stored at 25 °C under a relative humidity of 55% for 24 h [7].

#### 2.9.2. Determination of the Weight Loss Ratio, Hardness and Chewiness of Blueberries

Weight of blueberries was recorded at 1, 4, and 7 d during storage, and the percentage of weight loss was calculated every three days. Hardness and chewiness of blueberries were evaluated according to the texture profile analyses (TPA) method by using a TA-XT express texture analyzer (TA-XT2, Stable Micro Systems Ltd., Haslemere, UK) with a P/36R cylindrical probe. The speed was 1 mm/s, trigger was 5 g and compression was set to create 70% strain.

#### 2.9.3. Determination of the Color of Fresh-Cut Bananas

Color of samples was evaluated by using a colorimeter (Konica Minolta, Chroma Meter, CR400, Tokyo, Japan). The instrument was standardized using standard white plates, and the CIE L*a*b* color space values were registered. The color parameters of origin sample were recorded as *L_0_*, *a_0_*, *b_0_*, and the color parameters of sample after 24 h storage were recorded as *L*, *a*, *b*. The changes of color parameters were calculated as: ΔL* = *L* − *L_0_*, Δa* = *a* − *a_0_*, Δb* = *b* − *b_0_*.

### 2.10. Statistical Analysis

Data was expressed as mean ± standard deviation (SD) from triplicate determinations. The analysis of variance (ANOVA) was performed with SPSS (version 26.0 for Windows, SPSS Inc., Chicago, IL, USA) and the Tukey test was used to determine significant differences between means (*p* < 0.05).

## 3. Results and Discussions

### 3.1. Effect of TP on the Physical Properties of Potato Starch-Based Film

The physical properties of film are critical for its further potential application in different areas, which can provide important reference to judge whether the film has commercial value [20]. In the present study, the thickness, moisture content, color, light transmittance, tensile properties (including the tensile strength and elongation at break) and thermal properties were adopted to evaluate the physical properties of films with different concentrations of TP.

#### 3.1.1. Thickness, Moisture Content and Color

**Figure 1:**
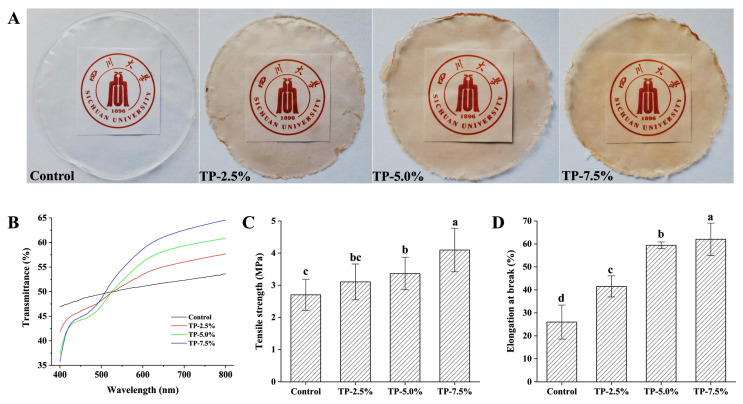
(**A**) graphs of potato starch-based film, (**B**) light transmittance, (**C**) tensile strength and (**D**) elongation at break of potato starch-based film incorporated with different concentration of TP.

As shown in Table 1, the addition of different concentrations of TP did not cause the obvious effect on the thickness of film. Commonly, the thickness of films is related to consumer acceptance when the films are used for food packaging [21]. Besides, the addition of TP decreased the moisture content of film. It has been reported that phenolic hydroxyl groups of phenols can competitively grab the water molecules in the film-forming system. Commonly, plasticizers, such as glycerol, are used to keep the moisture of film in the film formation process [22]. The addition of TP might combine with water molecules by hydrogen bonds, thus interfering with the binding of glycerol to water. It has also been reported that adding polyphenols can limit the interactions of the polysaccharides and glycerol with water, thus decreasing the moisture content of films [23]. In addition, TP endowed the film with a reddish-brown color, as shown in Figure 1A, which was attributed to the color of TP itself. As shown in Table 1, the color of the films was largely dependent on the addition levels of TP, and increased with the increasing of TP concentration in film. The L* value decreased, and the a*, b* and Δ*E* values increased, which indicated that the film with higher additions levels of TP was darker, redder and yellower.

#### 3.1.2. Light Transmittance and Tensile Properties

As shown in Figure 1B, the addition of TP could change the light transmittance of film, especially in the range of 550 to 800 nm. In the range of 550 to 800 nm, TP increased the light transmittance of film. It might be attributed to that the reddish-brown color of film, which was more conducive to the transmission of high wavelength light. Besides, tensile properties (tensile strength and elongation at break) of film was analyzed. As shown in Figure 1, TP increased the tensile strength (Figure 1C) and elongation at break (Figure 1D) of film, which indicated that TP at the testing concentration levels could improve the tensile properties and increase resistance towards stress without decreasing extensibility. It has been reported that the tensile properties of starchy film mainly depend on the random collision, winding and short-term retrogradation among starch chains [24]. The binding conformation among starch molecular chains is closely related to the strength of films, and the formation of a double helix structure can hinder the relative displacement between chains, leading to the decrease of elongation at break [2]. Combined with some relative studies, some water-soluble polyphenols can interact with starch molecules to interfere with the winding and binding between starch molecules, further change the structure of starchy film and tensile properties [3,5].

#### 3.1.3. Thermal Properties

**Figure 2:**
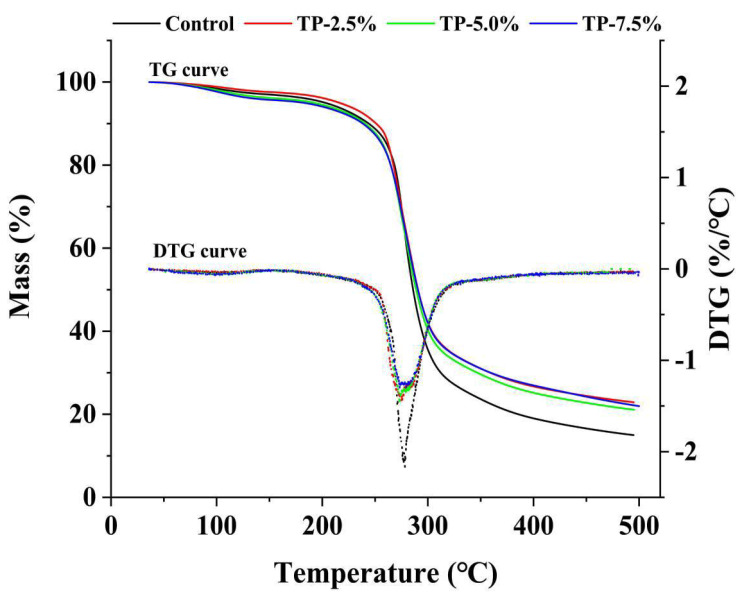
Thermogravimetric (TG) and derivative thermogravimetric (DTG) curves of potato starch-based film incorporated with different concentrations of TP.

As shown in Figure 2, the thermogravimetric loss of starch-based film mainly occurred in two stages. The first stage (<120 °C) was mainly caused due to the vaporization of bound water in film, which was associated with a slight weight loss. The second stage (200 to 350 °C) was mainly caused by the pyrolysis of phenolic compounds, the extensive thermal degradation of starch chain and so on, which presented a rapid weight loss in the TG curve, as well as a sharp and well-defined peak in the DTG curve [9]. According to the TG curve and DTG curve in Figure 2, the addition of TP increased the mass of the final products of film cracking and decreased the maximum cracking rate of film, which indicated that TP could increase the thermal stability of film. It has been reported that the effect of polyphenols on the thermal properties of film mainly affected the film structure [25]. All above results indicated that TP could change the physical properties of potato starch-based film (especially the elevate of tensile properties and thermal properties), which suggested the potential of TP for preparing potato starch-based film. Therefore, the barrier properties and functionality of this film were further evaluated.

### 3.2. Effect of TP on the Barrier Properties and Functionality of Potato Starch-Based Film

#### 3.2.1. Water Vapor Permeability (WVP) and Oxygen Permeability (OP)

**Figure 3:**
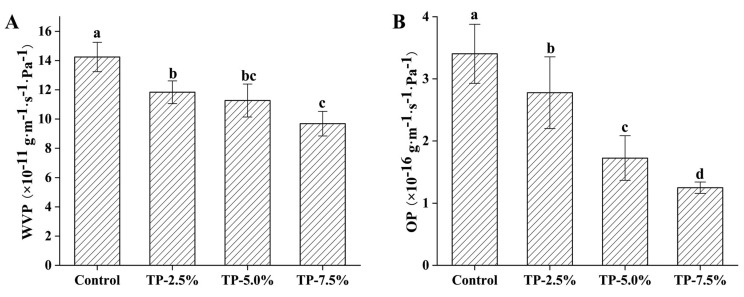
(**A**) water vapor permeability (WVP) and (**B**) oxygen permeability (OP) of potato starch-based film incorporated with different concentrations of TP.

The barrier properties of starch-based films related to permeability of water vapor and oxygen [2]. Commonly, the lower WVP and OP value indicates the stronger water vapor and oxygen barrier properties of film [26]. As shown in Figure 3, the addition of TP decreased the WVP (Figure 3A) and OP (Figure 3B) of films, especially higher dose of TP group, which indicated that TP could increase the barrier properties of film to water vapor and oxygen. It has been reported that the interactions between polyphenols and polysaccharides may lead to a more compacted structure for films, resulting in decreased permeability [27]. Commonly, water and oxygen in the air are closely related to food spoilage [28]. The enhancement of water vapor and oxygen barrier capacity of starch-based films is conducive to their use in the field of food packaging and preservation.

#### 3.2.2. Antioxidant Activity

**Figure 4:**
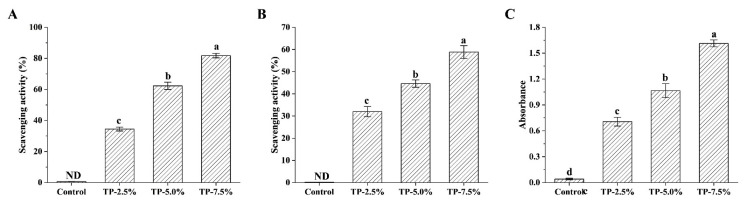
Antioxidant activity of potato starch-based film incorporated with different concentration of TP. (**A**) ABTS radical scavenging activity, (**B**) DPPH radical scavenging activity, (**C**) reducing power.

The antioxidant activity of film was evaluated and presented in Figure 4. Starch-based film without TP had no antioxidant activity (Figure 4A: ABTS free radical scavenging capacity, Figure 4B: DPPH free radical scavenging capacity, Figure 4C: reducing power), whereas the addition of TP obviously increased the antioxidant activity of starch-based film. Commonly, TP has been used as a natural and safe antioxidant for food processing and storage, which can effectively inhibit the quality deterioration of food [11]. Therefore, when the film was incorporated with TP and used for food packaging, TP in the film preferentially reacted with oxygen, due to its strong antioxidant activity, which might reduce the permeability of oxygen to a certain extent and delay the oxidation of food.

### 3.3. Interaction between Potato Starch and TP and the Structure of Film

The intertwining and binding of starch molecules constitute the structure of starch-based film. The structure of film is closely related to its physical properties and barrier properties [24]. The addition of TP might interfere with the combination among starch molecules, thus changing the structure of starch-based film. Therefore, WAXD and FTIR were used to analyze the interaction between potato starch and TP; then, SEM was adopted to further observe the effect of TP on the microstructure of film.

#### 3.3.1. WAXD and FTIR

**Figure 5:**
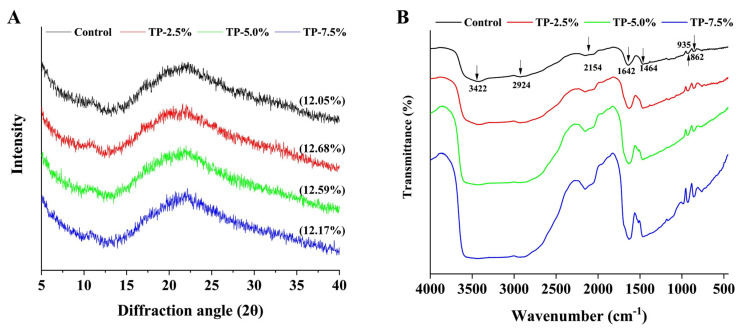
(**A**) wide-angle X-ray diffraction pattern, the value in the parenthesis represented relative crystallinity% (RC%). (**B**) Fourier transform infrared spectra of potato starch-based film incorporated with different concentration of TP.

According to the result of WAXD (Figure 5A), the peak position of diffraction peak was mainly located at 2θ~17° and 22°; these two diffraction peaks were attributed to the retrogradation of B-type starch after gelatinization [9]. Besides, the addition of TP did not increase the peak position of diffraction peak and not significantly change the relative crystallinity, which indicated that the interaction between TP and starch molecules did not lead to new crystals. This result also showed that TP had good dispersibility in starch-based film [13]. Subsequently, FTIR was used to analyze the interaction among components in the film. As shown in Figure 5B, the addition of TP did not change the wave peak number of film in the FTIR spectrum. Commonly, a broad band appeared at 3600**–**3000 cm**^−^**^1^ in FTIR spectrum, associated with the O**–**H stretching; the absorption at 2926 cm**^−^**^1^ indicates the C**–**H stretching, the absorption at around 2200 cm**^−^**^1^ indicates the =C**–**H stretching, a sharp peak at around 1633 cm**^−^**^1^ is attributed to the **–**C=O stretching, and the region (1200**–**800 cm**^−^**^1^) is applied to the fingerprint structure area of polysaccharide [9]. With the increase of TP concentration in film, the stretching vibration peak of O**–**H was broader, indicating the increase of amount and strength of intermolecular hydrogen bonds in film [9,29]. Thus, all results indicated that the interaction between starch molecules and TP was driven by non-covalent force.

#### 3.3.2. SEM

**Figure 6:**
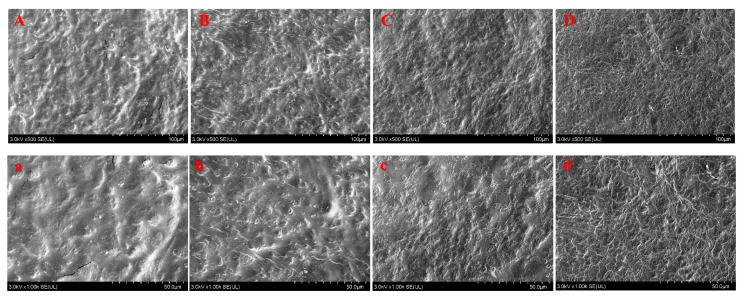
SEM images of potato starch-based film incorporated with different concentrations of TP. (**A**,**a**) control group, (**B**,**b**) 2.5% TP group, (**C**,**c**) 5.0% TP group, (**D**,**d**) 7.5% TP group. Capital letter denoted the amplification multiples of 500 times, and lowercase letters denoted the amplification multiples of 1000 times.

Microstructure of film was presented in Figure 6. The surface of film without TP was rough and uneven, showing many interlaced streaks (Figure 6A). Meanwhile, some cracks broken by the instrument electron beam were observed (Figure 6a). With the increase of TP concentration in film, the cross-linking degree of the film surface became higher, and the morphology became more compact and dense (Figure 6B/b to D/d), which might be the main cause for the improvement of tensile properties (TS and EB) and barrier properties (WVP and OP) of film. Especially in the high dose of TP group (7.5%), the surface of film exhibited more interlaced lines (Figure 6D/d). It has been reported that some polyphenol extracts can prevent amylose molecules to form double helix structure, thus interfering with the cross-linking between starch molecules [9,10,11,30]. Combined with above results (Figure 5 and Figure 6), TP could interact with potato starch chains via hydrogen bonds to form non-crystal complex, thus affecting the microstructure of film surface, so as to change its physical and barrier properties.

### 3.4. Application of Potato Starch-Based Film Incorporated with TP in Fruit Packaging

Proper physical properties, barrier properties and functionality endowed the film to show potential for fruit packaging [28]. In the present study, blueberries and fresh-cut bananas were employed as food models, to evaluate the effect of the film on the quality of blueberries and the browning degree of fresh-cut bananas during storage.

#### 3.4.1. Weight Loss Ratio, Hardness and Chewiness of Blueberries

Commonly, weight loss ratio, hardness and chewiness are usually employed to reflect the freshness and quality of fruit during storage [7]. As shown in Table 2, the weight loss ratio of blueberries in all groups gradually increased with the increase of storage time, which was due to the loss of moisture and the decomposition of organic matter in blueberries. During 7 days’ storage, the water in blueberries gradually volatilized through transpiration. Meanwhile, due to the breathing of fruit, blueberries needed consume the accumulated organic matter for providing the energy for their own physiological activities [7,31]. The blueberries without film lost more weight than those with film, which might be attributed to the fine barrier properties of film on water and oxygen (Figure 3). During the storage of blueberries, starch-based film inhibited the permeation of water and oxygen from the environment, and slowed down the transpiration and respiration of blueberries, so as to inhibit their weight loss. Besides, due to the weight loss and consumption of organic matter in blueberries, their hardness and chewiness gradually decreased during 7 days’ storage. However, all starch-based films inhibited the decline of weight, hardness and chewiness of blueberries compared with those without film. Meanwhile, film incorporated with TP could obviously slow down the decrease of weight, hardness and chewiness of blueberries. This was because TP improved the physical properties, barrier properties and functionality of starch-based film.

#### 3.4.2. Color and Browning Degree of Fresh-Cut Bananas

**Figure 7:**
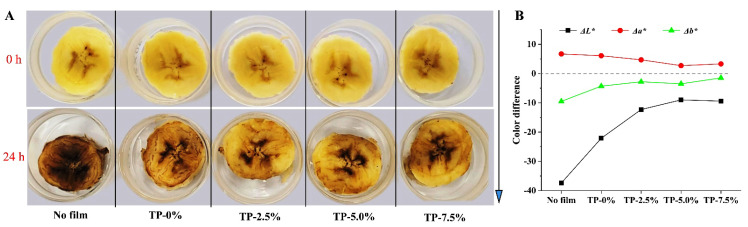
Effect of potato starch-based film incorporated with different concentrations of TP on the color of fresh-cut bananas. (**A**) graphs and (**B**) color parameters.

Enzymatic browning caused by polyphenol oxidase can make fresh-cut fruits darker, and reduce their sensory quality and commercial value [32]. As shown in Figure 7A, fresh-cut banana without film almost completely browned, whereas the enzymatic browning of the fresh cut banana with film was inhibited. Notably, film incorporated with TP exhibited a stronger capability to avoid the browning of banana, which might be attributed to its significant barrier properties on oxygen (Figure 3) and remarkable antioxidant activities (Figure 4). Meanwhile, according to the result of color parameters in Figure 7B, film incorporated with TP mainly inhibited the increase of absolute value of ΔL* for fresh-cut banana, which indicated that the addition of TP in film could delay darkening of fresh-cut banana.

## 4. Conclusions

In present study, tea polyphenols (TP) were incorporated into potato starch-based film. The addition of TP changed the physical properties (including thickness, moisture content, color, light transmittance, tensile properties and thermal properties) of film, especially the improvement of tensile properties (tensile strength and elongation at break). Meanwhile, film incorporated with a high dose (7.5%) of TP had a better barrier effect on water vapor and oxygen, as well as the strong free radical scavenging capacity and reducing power. Moreover, TP mainly combined with starch molecules by non-covalent forces, and affected the intertwining among starch molecules, thus making the film compact and dense. Furthermore, film incorporated with TP exhibited the significant capability to inhibit the decline of weight, hardness and chewiness of blueberries and the the enzymatic browning of the fresh-cut banana during storage. All present results suggest that TP could improve the properties and functions of potato starch-based film to various degrees, and have a potential application in fruit packaging and storage. Further studies are undergoing for the biodegradability of this film and its application for the packaging of meat.

## Figures and Tables

**Table 1:** Effect of tea polyphenols (TP) on the thickness, moisture content and color of potato starch-based film.

Sample	Thickness (mm)	Moisture Content (%)	L*	a*	b*	Δ*E*
control	0.089 ± 0.002 ^a^	10.27 ± 0.70 ^a^	33.18 ± 0.66 ^a^	−0.23 ± 0.01 ^d^	−1.20 ± 0.14 ^d^	-
TP-2.5%	0.087 ± 0.004 ^a^	9.68 ± 0.54 ^b^	28.59 ± 0.22 ^b^	0.77 ± 0.04 ^c^	1.83 ± 0.04 ^c^	5.41 ± 0.18 ^c^
TP-5.0%	0.083 ± 0.001 ^a^	9.45 ± 0.77 ^bc^	25.76 ± 0.65 ^c^	1.53 ± 0.13 ^b^	3.30 ± 0.17 ^b^	8.68 ± 0.60 ^b^
TP-7.5%	0.086 ± 0.002 ^a^	9.27 ± 0.63 ^c^	24.05 ± 0.75 ^d^	1.56 ± 0.03 ^a^	4.18 ± 0.14 ^a^	10.55 ± 0.52 ^a^

Each value is expressed as mean ± SD (n = 3). Different superscript letters in each vertical column denote statistically significant differences (*p* < 0.05).

**Table 2:** The weight loss ratio, hardness and chewiness of blueberries with and without film packaging during 7 days’ storage.

	Sample	Weight Loss Ratio (%)	Hardness (g)	Chewiness
1 d	-	-	995 ± 33	226 ± 29
4 d	No film	13.97 ± 3.13 ^a^	671 ± 47 ^e^	103 ± 37 ^d^
	Control	9.67 ± 1.42 ^b^	715 ± 61 ^d^	138 ± 23 ^c^
	TP-2.5%	9.59 ± 1.65 ^b^	803 ± 55 ^c^	147 ± 29 ^c^
	TP-5.0%	9.22 ± 2.11 ^c^	851 ± 73 ^b^	168 ± 36 ^b^
	TP-7.5%	7.89 ± 2.04 ^d^	906 ± 52 ^a^	174 ± 41 ^a^
7 d	No film	24.69 ± 3.81 ^a^	346 ± 29 ^d^	66 ± 26 ^d^
	Control	18.70 ± 1.98 ^b^	543 ± 33 ^c^	85 ± 11 ^c^
	TP-2.5%	17.53 ± 2.45 ^c^	590 ± 59 ^b^	89 ± 20 ^b^
	TP-5.0%	16.12 ± 1.98 ^d^	590 ± 46 ^b^	84 ± 18 ^c^
	TP-7.5%	15.93 ± 2.56 ^e^	631 ± 31 ^a^	101 ± 17 ^a^

## Data Availability

The data presented in this study are available on request from the corresponding author.
